# Supplementary material for: A Population-Based Cohort Study of the Association between Visual Loss and Risk of Suicide and Mental Illness in Taiwan
Source: Healthcare (Basel). 2023 May 18;11(10):1462. doi: 10.3390/healthcare11101462 (PMC10218556; doi:10.3390/healthcare11101462)
Supplement: Supplementary file 1 [file healthcare-11-01462-s001.zip › healthcare-2376096-supplementary.pdf]

Table S1. Abbreviation, ICD-9-CM, and definition

	Abbreviation	ICD-9-CM / Definition
**Stduy population:** Visual loss		369.3 - 369.4
Unqualified visual loss		369.3
Legal blindness		369.4
**Events:** Poor prognosis		Any of the listed
Mental disorders		≧ 3 outpatient visits or inpatient in 1 year
Anxiety		300
Depression		296.2-296.3, 300.4, 311
Bipolar		296.0, 296.4 - 296.8
Sleep disorders		307.4, 780.5
Posttraumatic stress disorder / acute stress disorder	PTSD / ASD	308, 309.81
Dementia		290.0 - 290.4, 290.8 - 290.9, 331.0
Eating disorders		307.1, 307.5
Substance-related disorders	SRD	291-292, 303.3, 303.9, 304 - 305
Psychotic disorders		295, 297-298
Autism		299.0
Other mental disorders		290 - 319 excluding listed above
Suicide		E950 - E959
All-caused mortality		000 - 999, E800 - E999
Suicide mortality		E950 - E959, Immediate and subsequent mortality
Non-suicide mortality		000 - 999, E800 - E949, E960 - E969
**Comorbidities:**		
Diabetes mellitus	DM	250
Hypertension	HTN	401-405
Renal disease		580-589
Hyperlipidemia		272
Thyrotoxicosis		242
Septicemia		003.1, 036.1, 038
Pneumonia		480 - 486
Chronic liver disease	CLD	571
Injury		800 - 999
Tumor		140 - 208
